# Risk factors of dengue fever in an urban area in Vietnam: a case-control study

**DOI:** 10.1186/s12889-021-10687-y

**Published:** 2021-04-07

**Authors:** Thang Nguyen-Tien, Duy Cuong Do, Xuan Luat Le, Thi Hai Dinh, Mats Lindeborg, Hung Nguyen-Viet, Åke Lundkvist, Delia Grace, Johanna Lindahl

**Affiliations:** 1 Department of Medical Biochemistry and Microbiology, Uppsala University, Uppsala, Sweden; 2 International Livestock Research Institute, Hanoi, Vietnam; 3 Infectious Diseases Department, Bach Mai hospital, Hanoi, Vietnam; 4 Section of Infectious Diseases, Department of Medical Sciences, Uppsala University, Uppsala, Sweden; 5 Center for Public Health and Ecosystem Research, Hanoi University of Public Health, Hanoi, Vietnam; 6 International Livestock Research Institute, Nairobi, Kenya; 7 Department of Clinical Sciences, Swedish University of Agricultural Sciences, Uppsala, Sweden

**Keywords:** Dengue fever, Dengue risk factors, Case-control, Urban setting, Vector-borne disease, Re-emerging diseas

## Abstract

**Background:**

Dengue is a mosquito-borne flavivirus present in many metropolitan cities of tropical countries.

**Methods:**

During and after the dengue season (September 2018 to January 2019), we conducted a case-control study in order to determine the risk factors for dengue fever in Hanoi city, Vietnam. 98 dengue patients and 99 patients with other acute infections, such as Hepatitis B virus infection, were recruited at Department of Infectious Disease of Bach Mai national hospital in Hanoi. Patients were interviewed using a structured questionnaire covering demographic, housing, environmental factors and knowledge, attitude, and practice on dengue prevention and control. Univariate analysis and multivariable logistic regression were used to determine the risk factors of dengue status.

**Results:**

The mean score of knowledge items and practice items was only 7.9 out of total 19 points and 3.9 out of total 17 points, respectively. While the mean score of attitude items was 4.8 out of total 6 points. Multivariable logistic regression indicated that older patients had lesser risk of getting dengue infection as compared to younger adults aged 16–30, and patients living in peri-urban districts were less likely to suffer of dengue fever than patients living in central urban districts (OR = 0.31; 95% CI 0.13–0.75). This study could not find any association with occupation, water storage habit, knowledge, attitude, or practice on dengue prevention.

**Conclusions:**

All patients had a relatively low level of knowledge and practice on dengue prevention and control. However, the attitude of the participants was good. We found that age group and living district were the risk factors correlated with the dengue status. Communication programs on raising dengue awareness should be repeated all year round and target particular groups of adolescents, younger adults, landlords and migrants from other provinces to improve their knowledge and encourage them to implement preventive measures against dengue fever.

**Supplementary Information:**

The online version contains supplementary material available at 10.1186/s12889-021-10687-y.

## Introduction

Dengue virus is one of the rare arboviruses that have fully adapted to humans and does no longer require an animal reservoir for transmission [1, 2]. This arbovirus transmits through mosquito vectors, primarily by *Aedes aegypti* and secondarily by *Ae. albopictus*. Dengue fever (DF) is known as the most widespread mosquito-borne disease globally, with approximately four billion people considered to be at risk [3]. Its incidence has increased 30-fold over the past five decades [4]. In 2010, there were 390 million people estimated to be infected by dengue virus, of which 96 million manifested clinically [5]. According to WHO, the number of dengue cases has increasing sharply and reached 4.2 million in 2019 [6]. Dengue is prevalent in Southeast Asia and the Pacific, which are hot spot areas for mosquito-borne diseases [7]. These regions account for around 75% of known global dengue morbidity and mortality and Vietnam is one of the countries suffering the highest burden [4, 7, 8].

In Vietnam, dengue virus is the most common flavivirus in rural and urban areas across the country [9, 10]. However, the virus circulates mainly in cities where the vectors easily find clean water for breeding [10]. According to the statistics from the World Health Organization (WHO) in Vietnam, dengue infections have increased from 105,370 cases in 2009 to 184,000 cases in 2017 [11]. Hanoi city, which is situated in Northern Vietnam, has a sub-tropical climate with four distinct seasons. Before 2008, Hanoi had nine urban and five peri-urban districts. After the city expanded its territory by merging some areas of other provinces, the new Hanoi has increased to twenty-nine districts and one town. It is a populous metropolis with around eight million people. In the past, Hanoi was considered to have a low incidence of dengue infection [12], however, the morbidity of dengue has increased during recent decades with outbreaks occurring in more frequent cycles. The dengue season in Hanoi begins in June, peaks in October, and decreases from December [12–15]. *Aedes* mosquitoes appears less active in the winter from November to February and then starts increasing its population when the summer comes [16]. Nevertheless, one recent study conducted in March 2018 found the dengue vectors, both mosquitoes and larvae, active also during the low season of dengue [17], which indicates a potential risk of dengue infections at any time of the year in Vietnam. With favorable conditions of weather, a dense human population and a rapid urbanization, Hanoi is at present an endemic area of DF. In this city, incidence has been increasing dramatically in the past decades, with the two largest outbreaks being recorded in 2009 and 2017 [14, 15, 18].

This study aimed to identify risk factors of DF in Hanoi city to generate more data on factors associated with dengue transmission in a populous metropolitan area. Its findings will help to propose appropriate interventions for dengue prevention and control programs.

## Methods

### Study design and setting

A case-control study was conducted during and after the 2018 season of dengue (from September 2018 to January 2019), at the Department of Infectious Diseases of Bach Mai, a large national hospital of Vietnam located in Hanoi city.

### Research subjects

In-patients and out-patients who were receiving treatment at the Department of Infectious Diseases were asked for their willingness to participate in the study. They were eligible if they were living in Hanoi city. If the patients were under 18 years old, the research group asked for permission from the guardian to join the study.

### Definitions

The criteria for DF confirmation was based on the dengue case definition in Decision 458/QD-BYT and its guideline issued by the Vietnam Ministry of Health in 2011. The content of the guideline includes the clinical and sub-clinical characteristics of DF; diagnosis and treatment of DF applying for all healthcare facilities in Vietnam. Patients, suspected for DF on clinical symptoms, were confirmed by a rapid test for dengue virus NS1 antigen during the first five days, or dengue-specific IgM after the fifth day of the disease [7, 19].

The case group included in-patients who were diagnosed for dengue and confirmed positive by the rapid tests for NS1 antigen or IgM antibodies. If the rapid test result was positive for NS1 antigen and negative for IgM, or vice versa, the diagnosis of the treating doctor was used to classify as a confirmed case or a negative case. The control group included out-patients or in-patients who were not diagnosed for DF and confirmed negative by the rapid test according to the case definition above. The interviewer did not record the information if the patient was in-patient or out-patient. No matching was performed.

The districts of participants living were categorized into three different areas: central urban, which comprises the inner districts of old Hanoi where no livestock is kept; peripheral that are newly expanded districts of new Hanoi where some livestock are kept, and peri-urban that comprises suburban districts where many livestock are kept.

### Sample size and sampling technique

By applying the formula for calculating the sample size of an unmatched case control study with the aim to be able to identify a risk factor with 20% difference between the different groups, as described by Fleiss et al. [20], 91 patients in each group were needed for this study. We increased this by 10% and intended to recruit 100 dengue cases and 100 dengue negative control cases. While it would have been good to be detect smaller differences between groups, we were limited by the access of patients. Finally, 98 cases and 99 control patients participated in the study.

### Data collection, instrument and Cronbach alpha

The questionnaire was developed using inputs from two infectious disease and epidemiology experts and piloted at the hospital with five patients (the data from these patients were excluded in the final analysis) for the suitability. Cronbach Alpha test was conducted and shown below with the acceptable score of each component for the questionnaire. Subsequently, the questions were entered into a Google form and a tablet was used by trained data collectors. The patients were interviewed during 15–25 min on demographic, household information, as well as knowledge, attitudes and practices (KAP) with regard to mosquitoes and DF. Cronbach alpha was used to test the internal consistency of the KAP components.

### KAP scoring

The questionnaire was designed with a set of multiple-choice or single-choice questions (Supplementary Material 1). Scores of one or zero were given to the correct and incorrect responses, respectively. Participants who achieved higher scores were assumed to have better knowledge, attitude or practices on DF.

#### Knowledge about dengue fever

The knowledge about DF was assessed by asking 8 questions (22 items) with a total score ranging from 0 to 19 points (Table 1).

**Table 1:** Scoring of knowledge items

Questions	Items	Score
a)	List the typical symptoms of dengue fever	
	1. Don’t know	0
	2. Joint and muscle pain	1
	3. Headaches, pain behind the eyes	1
	4. High fever continuously within 2–7 days (higher 39 degrees)	1
	5. Epistaxis	1
	6. Heamorrhagic under skin	1
	7. Stomachache, vomiting	1
	8. Bleeding gum	1
	**Min - Max**	**0–7**
b)	9. How does dengue virus transmits to people?	
	• Don’t know	0
	• Mosquito bites	1
	**Min - Max**	**0–1**
c)	10. Which type of mosquito is spreading dengue fever	
	• Don’t know	0
	• Stripe Mosquito (*Aedes aegypti*)/Tiger Mosquito (*Aedes albopictus*)	1
	• *Culex* Mosquito	0
	**Min - Max**	**0–1**
d)	11. The biting behavior of dengue mosquitoes	
	• Don’t know	0
	• Daylight	0
	• Night	0
	• Early in the morning and in the evening before dusk.	1
	**Min - Max**	**0–1**
e)	12. The breeding season of dengue mosquitoes	
	• Don’t know	0
	• Mainly in the rainy season	1
	• Mainly in the dry season	0
	• Both of seasons	0
	**Min - Max**	**0–1**
f)	List the breeding sites of dengue mosquitoes	
	13. Don’t know	0
	14. Water-filled jars, tanks	1
	15. Water-filled vases	1
	16. Water- filled used tires	1
	17. Garbage containing water	1
	**Min - Max**	**0–4**
g)	18. Can dengue fever be prevented?	
	• Yes	1
	• No	0
	• Don’t know	0
	**Min - Max**	**0–1**
h)	List the methods to prevent dengue fever	
	19. Don’t know	0
	20. Eliminate mosquito	1
	21. Eliminate the larvae	1
	22. Avoid mosquito bites	1
	**Min - Max**	**0–3**

#### Attitude on dengue fever

This part consisted of six questions in order to investigate the respondent’s opinion whether they agree/ don’t know/ do not agree. There was only one correct answer which was given 1 point. Other answers were given 0 point. The score of the attitude on DF ranged from 0 to 6 points (Table 2).

**Table 2:** Scoring of attitude items

Questions	Items	Score
a) a) a) a)	1. Dengue fever is a dangerous disease	
	• Agree	1
	• Do not agree	0
	• Don’t know	0
	**Min - Max**	**0–1**
b)	2. Mosquitoes play an important role in transmitting human diseases.	
	• Agree	1
	• Do not agree	0
	• Don’t know	0
	**Min - Max**	**0–1**
c)	3. The best measure to prevent dengue fever is to eliminate the breeding sites of mosquitoes.	
	• Agree	1
	• Do not agree	0
	• Don’t know	0
	**Min - Max**	**0–1**
d)	4. Children should be protected from mosquito bites.	
	• Agree	1
	• Do not agree	0
	• Don’t know	0
	**Min - Max**	**0–1**
e)	5. Household can spray anti-mosquito products/fogging by themselves without health staffs/ community.	
	• Agree	0
	• Do not agree	1
	• Don’t know	0
	**Min - Max**	**0–1**
f)	6. The responsibility of people’s health protection belongs to authority and health sector, not mine.	
	• Agree	0
	• Do not agree	1
	• Don’t know	0
	**Min - Max**	**0–1**

#### Dengue preventive practices

Preventive practices for DF was measured by using a set of 5 questions (15 items) on home behavior. The score of preventive practices on DF ranged from 0 to 17 points (Table 3).

**Table 3:** Scoring of practice items

Questions	Items	Score
a)	Preventive practices used to prevent mosquito bites at home	
	1. Don’t use any measures	0
	2. Wear long sleeves	1
	3. Use mosquito repellent creams/ liquid	1
	4. Use mosquito nets	1
	5. Use mosquito incense/coils	1
	6. Use mosquito racket	1
	7. Cover water storage	1
	8. Clean garbage having water	1
	9. Pruning the trees	1
	10. Remove standing water inside/outside house	1
	11. Spraying	1
	**Min - Max**	**0–10**
b)	12. Time of using bed net	
	• All the time (day and night)	2
	• Only during the day	1
	• Only during the night	1
	• Don’t use	0
	**Min - Max**	**0–2**
c)	13. Frequency of cleaning up water containers	
	• Weekly	2
	• Don’t have water containers/tanks	1
	• Monthly/1–2 times per year/Rarely/Never	0
	**Min - Max**	**0–2**
d)	14. Using fish for larva elimination	
	• Yes	2
	• Don’t have water containers/tanks	1
	• No	0
	**Min - Max**	**0–2**
e)	15. Using anti-mosquito spraying in your house	
	• Yes	1
	• No	0
	**Min - Max**	**0–1**

### Data analysis

Data was imported to Excel then transferred to SPSS for analysis. The associations between categorical variables were tested using Pearson’s chi-square or Fisher’s exact tests, while the mean of continuous variables without normal distribution between groups were compared by Mann-Whitney test. Logistic regression analysis was used to identify the risk factors of dengue infection. Initially, all factors were tested in an univariable logistic regression model. Then determinants with *p*-values less than, or equal to 0.25 (as previously used in another studies [21, 22]) and suspected confounders (including gender, age, education, marital status, occupation, average income, having chronic diseases, district where the patient lives, area of living, livestock keeping, storing water in the tank without lid, knowledge and attitude) and determinants found to be risk factors in the literature review (including storing water in the buckets/barrels and practice) were further analyzed by multivariable analysis with dengue status as the dependent variable. Confounders were explored by comparing the difference between the adjusted odds ratio in multivariable analyses and the crude odds ratio in univariate analyses. In the next step, models were built by manual backward deletion of highly non-significant variables including education, marital status, occupation, having chronic diseases, area of living, livestock keeping, storing water in the tank without lid, storing water in the buckets/barrels but keeping the KAP score variables as the important predictors. Since the participants who were pupils/students with no income accounting for a large proportion, we removed the average monthly income in the model to minimize the bias. Eventually, the determinants in the final model included gender, age, district where the patient lives, knowledge, attitude and practice scores. Odds ratios (OR) with 95% confidence interval (CI) were reported to present the association. *P-*values less than 0.05 were considered statistically significant. The analyses were also repeated with the scores calculated without weights, with each question contributing maximum 1 point, to see the impact on the final model. The statistical procedures were performed using IBM SPSS version 26.0 (SPSS, Chicago, USA).

## Results

One hundred and ninety-seven patients were recruited in the study including 98 dengue patients and 99 patients without dengue. Table 4 shows the demographic characteristic of the two groups. There was no significant difference in gender between the dengue and the control groups. Dengue patients were younger than patients without dengue (*p* < 0.001), with 63.3% of dengue patients being 16–30 years. Patients with higher education level, college or above, accounted for the majority of total participants (56.3%). There were more dengue cases among patients with higher education level as compared to patients with lower education level. Almost all patients were married (70.1%). Pupils/students (36.7%) and office workers (25.5%) were more likely to present with dengue infections. The average income in the dengue group was higher than in the control group (*p* < 0.001). Less dengue patients than control patients reported chronic diseases (*p* < 0.05). The chronic diseases reported included diabetes, cardiovascular, hypertension and hepatitis B.

**Table 4:** Demographic characteristics of participants

Demographic characteristics	***Dengue patients (%)*** ***N = 98***	***Control patients (%)*** ***N = 99***	***All (%)*** ***N = 197***	***P value***
***Gender*** ^***a***^				
Female	43 (43.9%)	53 (53.5%)	96 (48.7%)	0.225
Male	55 (56.1%)	46 (46.5%)	101 (51.3%)	
***Age group*** ^***a***^				
16–30	62 (63.3%)	27 (27.3%)	89 (45.2%)	**< 0.001**
31–45	20 (20.4%)	28 (28.3%)	48 (24.4%)	
46–60	15 (15.3%)	23 (23.2%)	38 (19.3%)	
> 60	1 (1%)	21 (21.2%)	22 (11.2%)	
***Education level*** ^***a***^				
High school and lower	32 (32.7%)	54 (54.5%)	86 (43.7%)	**0.003**
College and higher	66 (67.3%)	45 (45.5%)	111 (56.3%)	
***Marital status***^***b***^				
Single	42 (42.9%)	15 (15.2%)	57 (28.9%)	**< 0.001**
Married	56 (57.1%)	82 (82.8%)	138 (70.1%)	
Widowed	0	2 (2%)	2 (1%)	
***Occupation*** ^***a***^				
Office workers	25 (25.5%)	25 (25.3%)	50 (25.4%)	**< 0.001**
Farmer	5 (5.1%)	13 (13.1%)	18 (9.1%)	
Pupil /Student	36 (36.7%)	13 (13.1%)	49 (24.9%)	
Unemployed	2 (2%)	13 (13.1%)	15 (7.6%)	
Retired	2 (2%)	15 (15.2%)	17 (8.6%)	
Other	28 (28.6%)	20 (20.2%)	48 (24.4%)	
***Average income*** ^***c***^ ***(million VND/month)***				
Mean ± SD	11.9 ± 7.9	8.3 ± 4.5	10.2 ± 6.7	**< 0.001**
***Having chronic diseases***^***a***^				
Yes	10 (10.2%)	23 (23.2%)	33 (16.8%)	**0.024**
No	88 (89.8%)	76 (76.8%)	164 (83.2%)	

^a^Chi-square test, ^b^ Fisher exact test, ^c^ Mann-Whitney test

Table 5 indicates that most dengue patients were living in central and peripheral areas while the majority of control patients were from peri-urban areas (*p* < 0.01). The control group had a larger living area than the dengue group (*p <* 0.01). The number of people living with the dengue and control patients was similar with less than 4 persons on average. Few participants were keeping livestock and pets at home (5.1 and 19.3% respectively). Only few patients reported storing water in tanks without lids (5.2%) and buckets/barrels (13.3%). More than 70% of both groups had vegetation around their living space.

**Table 5:** Living conditions of participants

	***Dengue patients (%)***	***Control patients (%)***	***All (%)***	***P value***
***Living district*** ^***a***^				
Central urban	46 (46.9%)	34 (34.3%)	80 (40.6%)	**< 0.001**
Peripheral	41 (41.8%)	24 (24.2%)	65 (33%)	
Peri urban	11 (11.2%)	41 (41.4%)	52 (26.4%)	
***Number of people living with*** ^***c***^				
Mean ± SD	3.6 ± 1.7	3.8 ± 1.5	3.7 ± 1.6	0.314
***Area of living (m***^***2***^***)*** ^***c***^				
Mean (± SD)	54.8 ± 57.5	72.5 ± 69.9	64.1 ± 64.7	**0.004**
***Livestock keeping*** ^***b***^				
Yes	3 (3.1%)	7 (7.1%)	10 (5.1%)	0.331
No	95 (96.9%)	92 (92.9%)	187 (94.9%)	
***Pet keeping*** ^***a***^				
Yes	17 (17.3%)	21 (21.2%)	38 (19.3%)	0.612
No	78 (78.8%)	81 (82.7%)	159 (80.7%)	
***Storing water in the tank without lid*** ^***b***^				
Yes	2 (4.1%)	7 (7.4%)	9 (5.2%)	0.183
No	77 (97.5%)	87 (92.6%)	164 (94.8%)	
***Storing water in the buckets/barrels*** ^***a***^				
Yes	9 (11.4%)	14 (14.9%)	23 (13.3%)	0.652
No	70 (88.6%)	80 (85.1%)	150 (86.7%)	
***Vegetation*** ^***a***^				
Abundant vegetation	25 (25.5%)	24 (24.2%)	49 (24.9%)	0.966
Some vegetation	30 (30.6%)	32 (32.3%)	62 (31.5%)	
Little vegetation	18 (18.4%)	20 (20.2%)	38 (19.3%)	
No vegetation	25 (25.5%)	23 (23.2%)	48 (24.4%)	

^a^Chi-square test, ^b^ Fisher exact test, ^c^ Mann-Whitney test

**Fig. 1:**
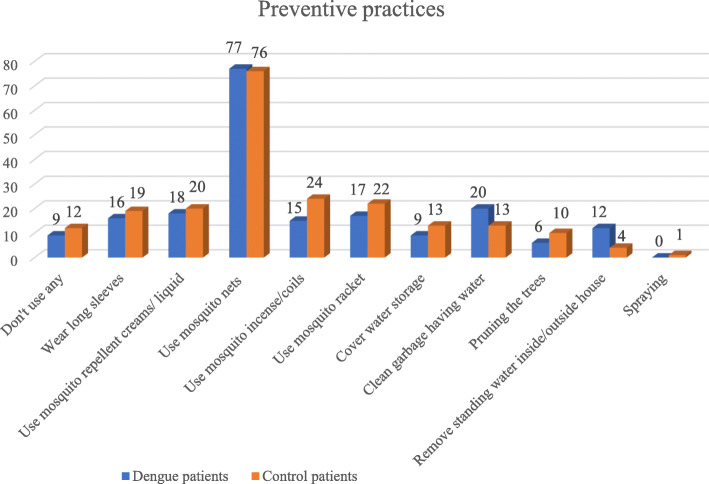
Number of patients that used protective practices at home to prevent dengue fever

Figure 1 reveals the number of patients using preventive practices at home to protect themselves from DF. Mosquito nets were the major preventive measure in both groups. Spraying around the house was used only by one control patient. Remarkably, there were 9 out of 98 dengue patients and 12 out of 99 control patients that did not use any measures at home to prevent DF.

Regarding to the Cronbach alpha, our results for each component are 0.75 for 22 knowledge items, 0.78 for 6 attitude items and 0.68 for 15 practice items, respectively.

The mean score on dengue knowledge among the dengue patients was higher than among the control patients (*p* < 0.001) (Table 6). Although dengue patients had a slightly better score on attitude and practice about DF as compared to the control group, these differences were not statistically significant (*p* > 0.05). However, Spearman’s rho test showed a positive correlation between the knowledge score and the practice score (*r =* 0.38 and *p* < 0.001).

**Table 6:** Knowledge, attitude and practice scores of the two groups

	***Dengue patients***	***Control patients***	***All***	***P value***
Knowledge score*				
Mean ± SD	8.7 ± 2.6	7 ± 3.3	7.9 ± 3.0	**< 0.001**
Attitude score*				
Mean ± SD	5.2 ± 0.8	4.5 ± 1.9	4.8 ± 1.5	0.059
Practice score*				
Mean ± SD	4 ± 2.4	3.8 ± 2.5	3.9 ± 2.4	0.305

* Mann-Whitney test

In Table 7, univariate analysis revealed that education, marital status, occupation, average income, having chronic diseases were associated with dengue status (*p <* 0.05). But these factors were eliminated in the final logistic regression model. Lower knowledge scores and attitude scores were also risk factors of getting dengue disease in the univariate analysis (*p <* 0.01). However, this was not statistically significant by the multivariate analysis (*p >* 0.05).

**Table 7:** Crude and adjusted OR in the logistic regression model

Risk factors	***Crude OR***	***CI 95%***	***P value***	***Adjusted OR***	***CI 95%***	***P value***
***Gender***						
Male	1	–	–	1	–	–
Female	0.68	0.39–1.2	0.176	1.58	0.81–3.06	0.18
***Age group***						
16–30 years old	1	–	–	1	–	–
31–45 years old	0.311	0.15–0.65	**0.002**	**0.43**	**0.19–0.95**	**0.038**
46–60 years old	0.284	0.13–0.63	**0.002**	**0.38**	**0.15–0.91**	**0.031**
Above 60 years old	0.021	0.003–0.16	**< 0.001**	**0.05**	**0.01–0.39**	**0.005**
***Education***						
High school and lower	1	–	–	–	–	–
College and higher	2.48	1.4–4.4	**0.002**			
***Marital status***						
Single	1	–	–	–	–	–
Married	0.24	0.12–0.48	**< 0.001**			
***Occupation***						
Office workers	1	–	–	–	–	–
Farmer	0.38	0.12–1.24	0.11			
Pupil/Student	2.77	1.19–6.43	**0.018**			
Unemployed	0.15	0.03–0.75	**0.021**			
Retired	0.13	0.03–0.64	**0.012**			
Other	1.4	0.63–3.1	0.4			
***Average income***	1.1	1.04–1.17	**< 0.001**	–	–	–
***Having chronic diseases***						
Yes	1	–	–	–	–	–
No	0.38	0.17–0.84	**0.017**			
***Living district***						
Central urban	1	–	–	1	–	–
Peripheral	1.26	0.64–2.47	0.49	1.35	0.64–2.84	0.43
Peri-urban	0.2	0.09–0.44	**< 0.001**	**0.31**	**0.13–0.75**	**0.01**
***Area of living***	0.99	0.99–1	0.074	–	–	–
***Livestock keeping***						
Yes	1	–	–	–	–	–
No	0.41	0.1–1.65	0.21			
***Storing water in the tank without lid***						
Yes	1	–	–	–	–	–
No	0.32	0.06–1.6	0.166			
***Storing water in the buckets/barrels***						
Yes	1	–	–	–	–	–
No	0.74	0.3–1.8	0.5			
***Knowledge score***	1.21	1.09–1.35	**< 0.001**	1.1	0.97–1.26	0.15
***Attitude score***	1.47	1.15–1.89	**0.002**	1.15	0.82–1.6	0.42
***Practice score***	1.05	0.94–1.18	0.415	0.97	0.84–1.13	0.71

* Using unweighted score, the final model identified the same independent variables as significant with unchanged adjusted OR

As compared to patients aged 16–30 years old, patients at older age of 31–45; 46–60 and above 60 years had a lower risk of 0.43 times (95% CI 0.19–0.95; *p <* 0.05); 0.38 times (95% CI 0.15–0.91; *p <* 0.05) and 0.05 times to get DF (95% CI 0.01–0.39; *p* < 0.01), respectively. Final multivariable logistic regression analysis indicated that patients living in peri-urban districts are 0.31 times less likely to get dengue infection than patients living in central urban districts (95% CI 0.13–0.75; *p* < 0.05). Unweighted scores as dependent variables identified the same predictors with unchanged adjusted OR. There was no difference in probability of getting dengue infection between patients living in central urban districts and peripheral districts (*p* = 0.43).

## Discussion

Our case-control study revealed that age and living area were associated with risk for dengue infection in the Hanoi metropolitan city. The results showed that older people had a lower risk for dengue infection as compared to younger people. In particular, people aged 31–45; 46–60 and above 60 years had 57, 62 and 95% lower risk, respectively, to get DF than patients aged 16–30. Those results are similar to earlier studies: People aged 15–34 years has been found to be the most infected age group in the dengue outbreaks in Hanoi city in particular and in Vietnam and Singapore in general [23–26]. This could be explained by the fact that the older people tend to pay attention on their health and have better health protective measures as compared to young adults, e.g. by sleeping under bed-nets at all time during the day. Also, it is possible that a larger proportion of the older people had been infected by dengue in the past and were immune. Immunity would mainly decrease the probability of getting the same serotypes of dengue virus [23]. In addition, young people may spend much more time on out-door activities, leading to higher risk of exposing with outside mosquitoes. Nevertheless, an earlier study in Singapore showed that the incidence rate for dengue was highest in the age group of 55 years and above, which is contradictory to our result [26]. An earlier systematic review and meta-analysis also revealed that the mean age of dengue patients reported after 2010 tends to be higher as compared to dengue cases reported before 2010 [27]. Therefore, it is suggested that health information, education and communication (IEC) program on dengue prevention and control should be disseminated to all age groups but focused on the adolescents and young adults.

Our study further indicated that people living in central districts have a 3.2 times higher risk to get an infection by dengue virus, as compared to people living in peri-urban districts. This is consistent with the epidemiological findings of Duong et al. [28] where 77.2% of the total dengue cases between 2006 and 2011 were concentrated to urban areas of Hanoi. Studies by Toan et al. [12] and Cuong et al. [23] showed similar results in that most cases of dengue were found in the inner districts of Hanoi. In Ho Chi Minh city, another big metropolitan area of Southern Vietnam, Raghwani et al. [29] also found that the more densely populated inner districts contributed significantly to DENV-1 transmission as compared to the suburban districts. Hanoi city is an economic center of Vietnam where migrants are populous. A lot of students and labor people from other provinces have been entering the central districts of Hanoi for living and working [30]. In addition to the low awareness on dengue prevention and control and limited resources, they may live in more unhygienic conditions that may increase the probability of creating breeding sites for dengue mosquitoes. This implies the higher risk of having DF and spreading the disease to neighboring people [23]. Our study also showed that students and office employees accounted for the highest percentage amongst the dengue patients. This result was in line with other studies conducted in Hanoi [23, 31]. Globally, 50% of the dengue outbreaks during 1990–2015 were recorded in urban areas, followed by 28.6% in rural areas, and 21.4% in both urban and rural areas [27]. Hence, living in urban areas is one of the driver of dengue dispersion due to the urbanization and huge population growth in the metropolitan regions [32], although it is reported that there was a movement of dengue morbidity from urban to rural settings [7]. The recommendation is that more communication campaigns should be organized in the central urban districts, targeted towards specific groups of landlords and their tenants.

Regarding other possible risk factors, this study suggested that gender was not a risk factor of having DF. However, it is noticeable that a study of Lien et al. [24] which was also carried out in Hanoi city, showed that males accounted for the majority of the dengue case in the 2011 outbreak. Studies by Guo et al. and Ler et al. also depicted that the incidence of getting dengue infection was significantly higher in males than in females [26, 27]. Therefore, it is hard to draw a conclusion on this association. It could also be that knowledge and education differs between gender, but this study had too low power to investigate that. KAP on dengue prevention and control are vital to measure the risk of getting DF. Interestingly, our findings indicated that individuals with dengue infection had better mean score of knowledge as compared to the control group (*p <* 0.001). This could be explained by the higher education in the dengue case group; or dengue patients may have improved their knowledge before the study started through the information by doctors, nurses or internet. Our study could not show any association between KAP score and dengue status, which was not in accordance with other studies in Vietnam [33]; in Malaysia [34, 35] and in Brazil [36], where the results confirmed a correlation between dengue infection and lacking of preventive measures such as not keeping the house and environment clean, storing water with uncovered containers, not wearing long sleeve clothes, or not using screen windows. In the era of information technology, it is easy to search for health information related to DF which is the most common mosquito-borne disease in Hanoi and Vietnam. In a KAP cross sectional study conducted in the same site in Hanoi during the 2017 dengue outbreak, the mean knowledge score of participants was 4.6 out of 19 points; lower than the mean score obtained in our study (7.9/19) [37]. It is understandable that people in Hanoi at present have a better knowledge on dengue prevention and control as compared to earlier studies [31]. However, knowledge alone does not produce individual behavior change [4]. In routine life, dwellers have numerous concerns rather than doing preventive measures for mosquito-borne diseases. Previous studies have proved that the behavior of water storage in uncovered containers was a high-risk factor for *Aedes* breeding [27, 36, 38]. Nevertheless, our study did not find any links between storing water without lid or in the buckets/barrels at home with the probability of being infected by dengue virus in the two patient groups. Since Hanoi is a capital city with a considerate speed of urbanization, the tap water system helps dwellers to change their storage habit and limit the mosquito breeding sites [23]. This may explain why few patients in our study had that behavior. However, Thang et al. [31] assumed that the breeding sites of mosquito could include public places like cemeteries with empty vases on the graves, temples and pagodas with many vases, the Bonsai or construction projects, and abandoned houses with stored garbage. Thus, our implication is that repeated messages on dengue prevention and control focusing on personal protections and environmental clean-up activities should be implemented at various time points of the year, not only when the dengue season starts in July.

In our study, livestock keeping was considered as a risk factor, as this has been found to be contributing to the risks of several vector-borne diseases [39, 40]. However, our results revealed no association between livestock keeping and dengue infections, perhaps due to the low number of individuals keeping livestock in this study. This finding was also demonstrated similarly in another study implemented in Hanoi city [17]. Further studies should be deployed to explore this issue in more detail, since urban livestock keeping is popular in many developing countries.

One strength of our study is that we used Cronbach alpha to determine the internal validity and reliability of KAP items in the questionnaire. The Cronbach alpha scores demonstrated satisfactory internal consistencies in our study. To our knowledge, this is first case-control study included all KAP components other than practice only to identify the risk factors of DF. Models were developed both with weighted and unweighted scoring system of KAP. As we mentioned above, the final models identified the same independent variables with the unchanged adjusted OR, indicating low bias of the unweighted estimates. However, this study still had several limitations that needs to be considered. Firstly, we could not match case and control patients who had different demographic characteristics since the sampling depended on patient availability and willingness to join. Due to the difficulties in recruiting controls, both inpatients and outpatients were included. Since sampling and interviewing was done only on one occasion, it is unknown if some control patients may later have been infected with dengue virus, but if diagnosed with DF, they would have been reclassified as a case. These limitations in control selection could lead to confounding and interaction of some variables including age, gender and knowledge. Nevertheless, we statistically controlled for possible confounders in the logistic regression model. Secondly, sampling was based on the selection of doctors at the Department of Infectious Diseases, and it is possible that this caused some selection bias. In order to minimize the bias, our study employed the updated national case definition of Vietnam Ministry of Health that all doctors in the Department of Infectious Disease were capable of and experienced in diagnosing the dengue case. Our research group also had a clearly documented selection procedure, and this was explained to all doctors before the start of the study. Thirdly, the patients may not be representative of the population of Hanoi because we excluded the children from 1 to 15 years old who is a vulnerable group suffering from DF. We only carried out the study in one hospital of Hanoi so that we could not infer these findings to the whole city’s population. Fourthly, the patients’ living place was not directly observed, leading to bias in their answers in some housing and environmental factors. Nevertheless, the interviewers were well trained and had experiences in data collection to get the validity of all participants’ responses.

In conclusion, our study found that younger adults aged 16–30 and people living in central urban areas have higher risk of getting dengue infection than older people and those living in peri-urban areas. KAP on dengue prevention and control and other demographic, environmental, housing variables were not related factors of dengue infection. Any IEC program on dengue prevention and control should be focused on specific groups of adolescents, younger adults, landlords and migrants and implemented many times of each year to improve the KAP of citizens.

## Supplementary Information

**Additional file 1.**

**Additional file 2.**

## Data Availability

The datasets used and/or analysed during the current study available from the corresponding author on reasonable request.

## Abbreviations

DF: Dengue fever
WHO: World Health Organization
KAP: Knowledge, attitude, practice
OR: Odds ratios
CI: Confidence interval
IEC: Information, education and communication
