# Correction: Risk factors of dengue fever in an urban area in Vietnam: a case-control study

**DOI:** 10.1186/s12889-022-13361-z

**Published:** 2022-06-02

**Authors:** Thang Nguyen-Tien, Duy Cuong Do, Xuan Luat Le, Thi Hai Dinh, Mats Lindeborg, Hung Nguyen-Viet, Åke Lundkvist, Delia Grace, Johanna Lindahl

**Affiliations:** 1 Department of Medical Biochemistry and Microbiology, Uppsala University, Uppsala, Sweden; 2 International Livestock Research Institute, Hanoi, Vietnam; 3 Infectious Diseases Department, Bach Mai hospital, Hanoi, Vietnam; 4 Section of Infectious Diseases, Department of Medical Sciences, Uppsala University, Uppsala, Sweden; 5 Center for Public Health and Ecosystem Research, Hanoi University of Public Health, Hanoi, Vietnam; 6 International Livestock Research Institute, Nairobi, Kenya; 7 Department of Clinical Sciences, Swedish University of Agricultural Sciences, Uppsala, Sweden

**Correction to: BMC Public Health 21, 664 (2021)**

**https://doi.org/10.1186/s12889-021-10687-y**

Additional File 2 has been removed from the original [1] publication because it contained confidential data.
