# Supplementary material for: Risk factors of dengue fever in an urban area in Vietnam: a case-control study
Source: BMC Public Health. 2021 Apr 7;21:664. doi: 10.1186/s12889-021-10687-y (PMC8028770; doi:10.1186/s12889-021-10687-y)
Supplement: Supplementary file 1 — Additional file 1. [file 12889_2021_10687_MOESM1_ESM.docx]

**RISK FACTOR FOR DENGUE FEVER**

**QUESTIONNAIRE**

Full name’s interviewee: ………………………………………………….

Address: ……………………………………………………………………………..

Phone number………………………………………………………………………

| **No** | **A. GENERAL INFORMATION** | | **Note** |
| --- | --- | --- | --- |
| A1 | Gender | 1. Male 2. Female. |  |
| A2 | Age (calculated by Gregorian Calendar) | _________ years old. |  |
| A3 | Highest level of completed education | 1. No education 2. Primary school 3. Secondary school 4. High school 5. Intermediate/ College 6. University/ Post-graduate. |  |
| A4 | Marriage status | 1. Single 2. Married 3. Separated/ Divorced 4. Widowed 5. Others: …...................................... |  |
| A5 | Main occupation | 1. Officer 2. Farmer 3. Pupil/Student 4. Unemployed 5. Retired 6. Others (specify): |  |
| A6 | Living area | ……………………………ward/commune…………………………………. district. |  |
| A7 | Duration of time you have lived there | ………month ……………. year |  |
| A8 | People who living with you  *(tick all that apply)* | 1. Alone 2. Spouse/ partner 3. Parents 4. Friends/ Colleagues 5. Children 6. Others (specify): |  |
| A9 | Average monthly income of your family | ………………………… VND |  |
| **No** | **B. INFORMATION OF DWELLING PLACE** | | **Note** |
| B1 | Current type of housing | 1. Brick construction 2. Temporary house 3. Old condominium 4. New condominium 5. Others: …………………… |  |
| B2 | Area of household (m²) | …………………………………… |  |
| B3 | Number of people in household | …………………………………people. |  |
| B4 | Is your family keeping livestock? | 1. Yes 2. No -> ***Go to B6.*** |  |
| B5 | If yes, what kind of livestock? Number of livestock?  (tick all that apply) | 1. Pig………………… 2. Cattle/cow………………. 3. Chicken…………… 4. Goose/Duck……………… 5. Others (specify): …………… |  |
| B6 | Is your family keeping pets? | 1. Yes 2. No -> ***Go to B8*** |  |
| B7 | If yes, what kind of pets?  Number of pets?  *(tick all that apply)* | 1. Dog…………………………. 2. Cat………………………….. 3. Song birds 4. Others (specify): ……………. |  |
| B8 | Do you store water in the house? | 1. Yes 2. No -> ***Go to B10.*** |  |
| B9 | How do your family store water?  *(tick all that apply)* | 1. Contain in closed tank/ underground tank 2. Contain the tank without lid 3. Contain the big jar 4. Contain in bucket, barrels 5. Others (specify): ……………… |  |
| B10 | Within yards of your house, are there any potential mosquito breeding sites? If yes, how many?  *(tick all that apply)* | 1. Don’t have any 2. Tires, ………………… 3. Cans, ………………… 4. Discarded boxes, ………… 5. Bottles, ……………….. 6. Dishes for feeding animals, ……… 7. Others (specify): ……………. |  |
| B11 | How was your family’s wastewater being processed? | 1. Discharged into the open sewer 2. Discharged into the river/lake nearby the house 3. Store wastewater to use for toilet 4. Others (specify): ………………… |  |
| B12 | Was your house flooded in the rainy season? | 1. Yes 2. No |  |
| B13 | Estimate the quantity of trees surrounding your house | 1. Abundant vegetation 2. Some vegetation 3. Little vegetation 4. No vegetation |  |
| **No** | **C. DISEASE INFORMATION** | | **Note** |
| C1 | Have you ever had dengue fever? | 1. Yes 2. Never -> ***Go to C5*** 3. Don’t remember -> ***Go to C5*** 4. Possibly but not sure |  |
| C2 | If you have been infected, how long ago? | …………………………………………… |  |
| C3 | Did you have dengue test at that time? | 1. Yes 2. No 3. Don’t remember. |  |
| C4 | What was the dengue test result? | 1. Negative with dengue virus 2. Positive with dengue virus 3. Don’t know/don’t remember. |  |
| C5 | Have you come to the endemic or outbreak areas in the last 6 months? | 1. Yes 2. No 3. Don’t know. |  |
| C6 | What were the first symptoms you have before going to hospital? | 1. Joint and muscle pain 2. Headaches, pain behind the eyes 3. High fever continuously within 2-7 days (higher 39 degrees) 4. Epistaxis 5. Bleeding under skin 6. Stomach ache, vomiting 7. Others (specify): …………………. |  |
| C7 | What did you do before going to hospital? | 1. Do nothing/ no treatment 2. Buy drugs without prescription 3. Go to private clinic/ other hospital 4. Others (specify): ……………….. |  |
| C8 | Did any family members have similar symptoms to yours? | 1. Yes 2. No 3. Don’t know |  |
| C9 | Did any neighbors surrounding your house have similar symptoms to yours? | 1. Yes 2. No 3. Don’t know. |  |
| C10 | Did any colleagues at your working place have similar symptoms to yours? | 1. Yes 2. No 3. Don’t know. |  |
| C11 | Do you have any chronic diseases?  *Tick all that apply* | 1. No disease 2. Diabetes 3. Cardiovascular disease 4. Hypertension 5. Don’t know 6. Others (specify): …………………. |  |
| **No** | **D. KNOWLEDGE ABOUT DENGUE FEVER** | | |
| D1 | Can you mention one or more symptoms typical of Dengue fever? | 1. Don’t know 2. Joint and muscle pain 3. Headaches, pain behind the eyes 4. High fever continuously within 2-7 days (higher 39 degrees) 5. Epistaxis 6. Bleeding under skin 7. Stomach ache, vomiting 8. Others (specify): ………………… |  |
| D2 | How does dengue fever transmit? | 1. Mosquito bite 2. Don’t know 3. Others (specify): …………… |  |
| D3 | What sort of mosquito spread Dengue fever? | 1. Stripe Mosquito (Aedes aegypti)/ Tiger Mosquito (Aedes albopitus) 2. Culex Mosquito 3. Don’t know. 4. Others (specify): ………….. |  |
| D4 | Can you tell the biting behavior of dengue mosquito? | 1. Daylight 2. Night 3. Day and afternoon, usually early in the morning and in the evening before dusk. 4. Don’t know 5. Others (specify): …………….. |  |
| D5 | Can you tell the breeding season of dengue mosquito? | 1. Don’t know. 2. Mainly in the rainy season 3. Mainly in the dry season 4. Both of seasons |  |
| D6 | Can you tell the breeding site of dengue mosquito?  *(Tick all that apply)* | 1. Water-filled jars, tanks 2. Water-filled vases 3. Water- filled used tires 4. Don’t know. 5. Others (specify): …………………… |  |
| D7 | Can dengue fever be prevented? | 1. Yes 2. No 3. Don’t know. |  |
| D8 | If yes, how to prevent dengue fever?  *(Tick all that apply)* | 1. Eliminate mosquito 2. Eliminate the larvae 3. Avoid mosquito bites 4. Don’t know 5. Others (specify): ……………….. |  |
| D9 | Have you ever heard about information on dengue fever? | 1. Yes 2. No. |  |
| D10 | Which sources of information you heard about dengue fever?  *(Tick all that apply)* | 1. Newspaper, magazines 2. Pictures/ posters/ 3. Television 4. Broadcast/Radio 5. Loudspeaker 6. Health staff 7. Internet 8. Friends/ Family relatives 9. Staff of the community organizations 10. Others (specify): ………………. |  |
| D11 | Currently, would you like to receive information about dengue fever? | 1. Yes 2. No. |  |
| D12 | If yes, which source of information you want to receive about dengue fever **most**? | 1. Newspaper, magazines 2. Pictures/ posters/ 3. Television 4. Broadcast/Radio 5. Loudspeaker 6. Health staff 7. Internet 8. Friends/ Family relatives 9. Staff of the community organizations 10. Others (specify): ………………. |  |
| **No** | **E. ATTITUDE ON PREVENTON OF DENGUE FEVER** | |  |
| E1 | Dengue fever is a dangerous disease | 1. Agree 2. Do not agree 3. Don’t know. |  |
| E2 | Mosquitoes play an important role in transmitting disease for human. | 1. Agree 2. Do not agree 3. Don’t know. |  |
| E3 | The best measure to prevent dengue fever is to eliminate the breeding sites of mosquitoes. | 1. Agree 2. Do not agree 3. Don’t know. |  |
| E4 | Children should be protected from mosquito bites. | 1. Agree 2. Do not agree 3. Don’t know. |  |
| E5 | Household can spray the anti-mosquito products by themselves without health staffs/ community. | 1. Agree 2. Do not agree 3. Don’t know. |  |
| E6 | Responsibility of people’s health protection belongs to authority and health sector, not mine. | 1. Agree 2. Do not agree 3. Don’t know. |  |
| **No** | **F. PRACTICE ON PREVENTON OF DENGUE FEVER** | |  |
| F1 | What do you do to prevent mosquito bites?  (tick all that apply) | 1. Don’t use any measures 2. Wear long sleeves 3. Use mosquito repellent creams/ liquid 4. Use mosquito nets 5. Use mosquito incense/coils 6. Use mosquito racket 7. Cover water storage 8. Clean garbage having water 9. Pruning the trees 10. Remove standing water inside/outside house 11. Others (specify): ……………… |  |
| F2 | During a day, when do you use bed net? | 1. All the time (day and night) 2. Only during the night 3. Only during the day 4. Don’t use. 5. Others (specify): ……………… |  |
| F3 | How often do you clean up water containers? | 1. Weekly 2. Monthly 3. 1-2 times per year 4. Rarely 5. Never 6. Don’t have water contaniners/tanks |  |
| F4 | Do you use fish for larva elimination? | 1. Yes 2. No 3. Don’t have water contaniners/tanks |  |
| F5 | Have you ever use anti-mosquito spraying in your house? | 1. Yes 2. No. |  |
| F6 | If yes, how long did the last spraying happen until now? | .....................................................months. |  |

***Thank you for your answer!***
